# Enhanced Regional Homogeneity and Functional Connectivity in Subjects With White Matter Hyperintensities and Cognitive Impairment

**DOI:** 10.3389/fnins.2019.00695

**Published:** 2019-07-03

**Authors:** Qing Ye, Xin Chen, Ruomeng Qin, Lili Huang, Dan Yang, Renyuan Liu, Bing Zhang, Feng Bai, Yun Xu

**Affiliations:** ^1^Department of Neurology, Affiliated Drum Tower Hospital, Jiangsu Key Laboratory for Molecular Medicine, Nanjing University Medical School, Nanjing, China; ^2^Jiangsu Province Stroke Center for Diagnosis and Therapy, Nanjing, China; ^3^Nanjing Neuropsychiatry Clinic Medical Center, Nanjing, China; ^4^Department of Radiology, Affiliated Drum Tower Hospital, Nanjing University Medical School, Nanjing, China

**Keywords:** white matter hyperintensities, cognitive impairment, regional homogeneity, functional connectivity, cognitive variety

## Abstract

**Objective:**

White matter hyperintensities (WMH) is an important cause of vascular cognitive impairment (CI). However, a considerable portion of individuals with WMH do not develop CI. The present study aimed to investigate distinctive regional brain activity and connectivity patterns in WMH subjects with and without CI, who displayed comparable WMH burden.

**Methods:**

Fourteen WMH subjects with CI, 16 WMH subjects without CI and 37 healthy subjects underwent multimodal MRI scans and neuropsychological tests. All WMH subjects displayed Fazekas grade 2 of WMH. Regional Homogeneity (ReHo) and functional connectivity (FC) patterns were identified based on resting-state functional MRI data.

**Results:**

No significant differences in WMH volume, the number of WMH lesions and brain volume were shown between the 2 WMH groups. In contrast, the WMH with CI group showed higher ReHo in bilateral superior parietal gyrus (SPG)/superior occipital gyrus (SOG) than the WMH without CI group. Compared with the WMH without CI group, the WMH with CI group also displayed higher FC of the left SPG/SOG with frontal regions, and higher FC of the right SPG/SOG with parietal regions. Furthermore, higher FC of the left SPG/SOG with frontal regions were significantly associated with less worse executive dysfunction in WMH with CI subjects, suggesting a compensatory effect.

**Conclusion:**

Higher local coherence of activities in the SPG/SOG and higher connectivity of the SPG/SOG with parietal and frontal regions are related to CI in WMH subjects. The findings provide novel insights into functional alterations underlying the cognitive variety in WMH subjects.

## Introduction

White matter hyperintensities (WMH), defined in the T_2_ weighted magnetic resonance imaging (MRI) representation, is widely common in elderly population. The prevalence of WMH increases remarkably with age and is as high as 72–96% in population over 60 years old (Longstreth et al., 1996; de Leeuw et al., 2001; Zhuang et al., 2018; Lampe et al., 2019). As a MRI marker of cerebral small vessel disease, the pathology of WMH generally reflects loss of axons and myelin, myelin pallor and gliosis (Gouw et al., 2011). These lesions are associated with lacunar infarction occurrence (Xu et al., 2018), and may disrupt white matter tracts or U-fibers that mediate cortical-subcortical or cortical-cortical connections, thus resulting in cognitive impairment (CI).

A large body of evidence shows that WMH causes vascular CI (Prins et al., 2005; Debette and Markus, 2010; Brickman et al., 2015), and WMH is associated with impairments in executive function and processing speed (Prins et al., 2005; Sudo et al., 2013). The baseline WMH burden was related with an increased risk of developing dementia, and the WMH progression was related with declines in global cognitive function and information processing speed (van Dijk et al., 2008). The progression of WMH correlated better with cognitive decline than did the baseline WMH burden (Schmidt R. et al., 2012). However, WMH is widely common in elderly population and not all subjects with WMH will develop CI. A recent study showed that WMH was detected in 77.8% healthy elderly between 60 and 82 years old (Lampe et al., 2019). Investigating the mechanisms underlying the link between CI and WMH may help to understand the cognitive heterogeneity in subjects with WMH.

Resting-state (fMRI) techniques have been increasingly utilized to investigate functional alterations related to the onset of CI in WMH. Recently, a study indicated that Regional Homogeneity (ReHo) in the left cerebellum and the middle cingulate cortex were significantly correlated with CI and executive function deficits respectively in subjects with both CI and WMH (Diciotti et al., 2017). Compared with WMH subjects with normal cognition, WMH subjects with CI displayed lower functional connectivity (FC) of posterior cingulate cortex with anterior cingulate cortex, temporal regions and frontal regions, and higher FC with specific temporal regions and parietal regions (Sun et al., 2011). Several other studies detected altered FC or brain activation patterns across frontal, parietal, temporal and occipital regions in WMH subjects with normal cognition (Lockhart et al., 2015; De Marco et al., 2017; Shi et al., 2017).

Most of the above researches were performed only in WMH subjects with normal cognition or WMH subjects with CI. These findings did not explain why only a portion of the population with WMH would develop CI. The present study recruited WMH subjects with CI, WMH subjects without CI and healthy subjects, and the 2 WMH groups had comparable WMH burden. We hypothesized that different ReHo and FC patterns would be shown between the WMH with and without CI groups and these functional alterations may be related to the mechanism of WMH-mediated CI.

## Materials and Methods

### Participants

The present study was carried out in accordance with the latest version of the Declaration of Helsinki, and approved by the Drum Tower Hospital Research Ethics Committee. Thirty-seven healthy subjects and 30 subjects with WMH (Fazekas grade 2) were recruited at the Drum Tower Hospital, Medical School of Nanjing University. All participants provided written informed consents and underwent multimodal MRI scans and a standardized diagnostic evaluation, including demographic data, medical history and an examination of neuropsychological status.

### Neuropsychological Examination

Global cognitive function was measured using a Montreal Cognitive Assessment (MoCA) and a Mini Mental State Examination (MMSE). WMH subjects with MoCA scores lower than education-adjusted norms (the cutoff was ≤ 19 for 1∼6 years of education, ≤24 for 7∼12 years of education and <26 for >12 years of education) were defined as the WMH with CI group (*n* = 14), and other WMH subjects were defined as the WMH without CI group (*n* = 16). All subjects underwent a neuropsychological battery test including Trail Making Tests (TMT)-A and B and Stroop Color and Word Tests A, B, and C (Stroop-A, B, and C). Three WMH with CI subjects failed to perform some of tests due to subjective unwillingness or hypopsia. The mental statuses were assessed with the *Structured Clinical Interview for Diagnostic and Statistical Manual of Mental Disorders*, Fourth Edition (DSM-IV) Axis I Disorders (SCID-I), the Hamilton Anxiety Scale (HAMA), and the Hamilton Depression Scale (HAMD).

### Inclusion and Exclusion Criteria

The inclusion criteria for WMH subjects were as follows: (1) age > 50 years, (2) the presence of Fazekas grade 2 of WMH on MRI images, (3) possible subjective complaints like memory impairment, postural instability, dizziness or depression. WMH were described as hyperintensities on FLAIR images, without cavitation. Fazekas grade 2 of WMH was defined as single lesions between 1 and 2 cm, areas of “grouped” lesions more than 2 cm in any diameter, and no more than “connecting bridges” between individual lesions (Pantoni et al., 2005).

Exclusion criteria were as follows: (1) a history of ischemic stroke with infarcts of more than 1.5 cm in diameter or cardiogenic cerebral infarction, (2) intracranial hemorrhage, (3) carotid artery stenosis (>75%) or coronary atherosclerosis heart disease, (4) other neurological disorders, such as Alzheimer disease (AD), Parkinson(ism), epilepsy and multiple sclerosis, (5) systemic disease, such as cancer, shock, anemia and thyroid dysfunction, (6) MRI contraindications, (7) prominent impairments of audition or vision.

### MRI Procedures

Magnetic resonance imaging scanning was performed using a 3 Tesla MR scanner (Achieva 3.0 T Ingenia; Philips Medical Systems, Eindhoven, Netherlands) with a 32-channel head coil at the Drum Tower Hospital, Medical School of Nanjing University. All subjects were told to relax, close their eyes and stay awake during scanning. Their heads were immobilized using belts and foam pads to minimize head motion, and their ears were occluded with earplugs. High-resolution T_1_-weighted sagittal images covering the whole brain were obtained by a 3D-magnetization prepared rapid gradient-echo sequence: repetition time (TR) = 9.8 ms; echo time (TE) = 4.6 ms; field of view (FOV) = 256 × 256 mm; acquisition matrix = 256 × 256; flip angle (FA) = 8°; thickness = 1.0 mm, gap = 0 mm; number of slices = 192. Resting-state functional images, including 230 volumes, were obtained by a gradient-recalled echo-planar imaging (GRE-EPI) sequence: TR = 2000 ms; TE = 30 ms; FOV = 192 × 192 mm; acquisition matrix = 64 × 64; FA = 90°; thickness = 4.0 mm; gap = 0 mm; number of slices = 35. Additionally, T_2_ FLAIR axial images were obtained with following parameters: TR = 4500 ms; TE = 344 ms; acquisition matrix = 272 × 272; FA = 90°; thickness = 1 mm; gap = 0 mm, number of slices = 200.

### WMH Segmentation and Quantification

**FIGURE 1:**
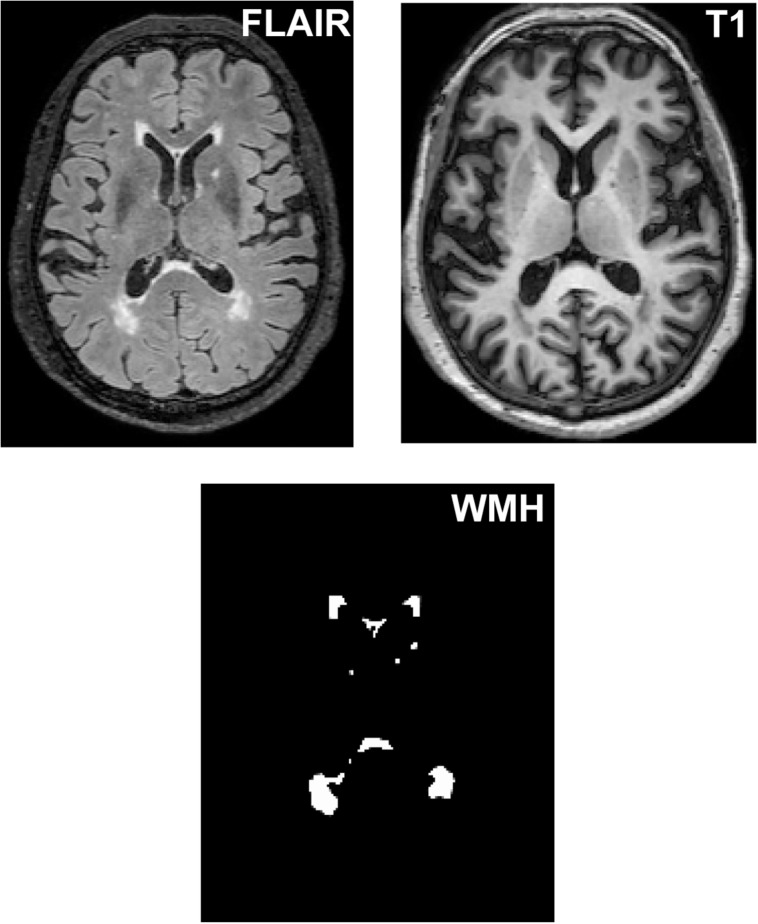
The segmentation of WMH lesions. WMH lesions were segmented and quantified from T_2_ FLAIR images and T_1_ images.

As shown in Figure 1, WMH volume was measured on T_1_-weighted and T_2_ FLAIR images using the lesion growth algorithm (Schmidt P. et al., 2012) as implemented in the LST toolbox version 2.0.15^1^ for Statistical Parametric Mapping software (SPM12^2^). First, the algorithm segments the T_1_ images into gray matter, white matter and cerebrospinal fluid. The information is then combined with the coregistered T_2_ FLAIR intensities to calculate lesion belief maps. By thresholding these maps with a pre-chosen initial threshold (*κ* = 0.30), an initial binary lesion map is obtained and is subsequently grown along voxels that appear hyperintense on the T_2_ FLAIR image. The result is a lesion probability map. It should be noted that the *κ*-value was determined through the visual inspection of the results by 3 experienced raters.

### Volume of Brain and Hippocampus

As described in our previous study (Ye et al., 2017), brain volume was assessed using the VBM8 toolbox for SPM12. First, the T_1_ images were segmented into gray matter, white matter and cerebrospinal fluid. Second, the segmented images were normalized to the MNI template using a non-linear and affine spatial normalization and re-sampled to a voxel size of 1.5 × 1.5 × 1.5 mm. Third, Jacobian modulation was applied to the segmented images, which could be incorporated to compensate for the effect of spatial normalization. Fourth, the extracted gray matter, white matter and cerebrospinal fluid sets were smoothed with an 8-mm full width at half maximum Gaussian filter to decrease the effects of individual variation in gyral anatomy and to increase the signal-to-noise ratio. Finally, gray matter volume, white matter volume, cerebrospinal fluid volume and whole brain volume were obtained in each subject.

Hippocampal atrophy is a well-established indicator for the early diagnosis of AD (Aisen et al., 2010; Jack et al., 2010), which is the most common type of dementia in the elderly population. To rule out the CI due to AD, the hippocampal volume was assessed. The hippocampus (left and right separately) was isolated using automated anatomical labeling implemented through the Resting State fMRI Data Analysis Toolkit 1.7 software^3^. Then, the hippocampal regions were interpolated to the same sizes, dimension and origins with T_1_ images. And a mean volume index of all voxels within the hippocampal region (left and right) was extracted for each subject. Finally, the hippocampal volume was obtained by multiplying the mean volume index by the size of each voxel (1.5 × 1.5 × 1.5 mm) and the number of voxels within the hippocampal region.

### Resting-State Functional Image Preprocessing

Functional MRI data were preprocessed using a toolbox for Data Processing and Analysis for Brain Imaging (DPABI) V2.3^4^. Owing to T_1_ equilibration effects, the first 10 volumes of the scanning session were discarded. The slice timing and realignment procedures were conducted to correct for the time differences in acquisition among slices within one volume, and the motion effects (Friston 24-parameter model) during scanning. A control subject was excluded due to head motion artifacts exceeding 2° in rotation or 2 mm in transition. The resulting images were spatially normalized into a standard stereotaxic space with a 12-parameter affine approach and an EPI template image, and then resampled to 3 × 3 × 3 mm voxels, and smoothed with a Gaussian kernel of 6 × 6 × 6 mm. Then, white matter signal, cerebrospinal fluid signal and 24 head motion parameters were removed as covariates of no interest. The resulting fMRI data were band-pass filtered (0.01–0.08 Hz), and the linear trend of time courses was removed. Finally, scrubbing was performed. Volumes with framewise displacement (FD) larger than 0.5 mm with prior 1 and later 2 volumes were deleted, and subjects with fewer than 4 min of remaining data (about 50% volumes) were excluded (Power et al., 2012; Satterthwaite et al., 2013; Chen et al., 2019; Guo et al., 2019). After exclusion, 33 control subjects, 14 WMH without CI subjects and 14 WMH with CI subjects remained.

### ReHo Analysis

ReHo analysis was performed without smoothing using a toolbox for DPABI V2.3. According to a previous study (Zang et al., 2004), individual ReHo maps were obtained by calculating the Kendall’s coefficient concordance of the time series of a given voxel with those of its nearest neighbors (26 voxels) in a voxel-wise manner. To improve the normality and reliability of ReHo value across subjects (Zuo et al., 2013), all individual ReHo maps were standardized into ReHo *z*-value by subtracting the average voxel-wise ReHo obtained for the entire brain, and then dividing the resultant value by the standard deviation. Finally, generated ReHo maps were spatially smoothed with a Gaussian kernel of 6 × 6 × 6 mm.

### FC Analysis

Regions showing significant difference of ReHo between the WMH with CI group and the WMH without CI group served as seeds for FC analysis. For each subject, a mean time series of each seed region was extracted as a reference time course. Pearson cross-correlation analysis was conducted between the reference time course and time course of each voxel in the brain. Then, a Fisher’s *z*-transformation was used to improve the normality of the correlation coefficients $z=0.5\times \ln\frac{1+r}{1-r}$. Finally, the individual FC maps of each region showing significant group difference of ReHo were obtained.

### Statistical Analysis

#### Demographic, Neuropsychological, and Volume Data

A one-way analysis of variance (ANOVA) was performed in the analyses of age, education, volume data and mean FD with significance at *P* < 0.05 among the control group, the WMH without CI group and the WMH with CI group. χ^2^ test was applied in the analysis of gender among the three groups. Because neuropsychological data was non-normal distribution, the Kruskal–Wallis test was applied in the analyses of neuropsychological data with significance at *P* < 0.05 among the three groups. The SPSS 19.0 software (SPSS, Inc., Chicago, IL, United States) was employed in these statistical procedures.

#### ReHo and FC Data

The ReHo or FC differences among the three groups were analyzed by applying a voxel-wise one-way analysis of covariance (ANCOVA), controlling for age, gender, years of education and mean FD (using DPABI V2.3). The thresholds were set at a corrected *P* < 0.01, determined by Monte Carlo simulation for multiple comparisons (voxel-wise *P* < 0.01), and FWHM will be estimated to determine the threshold of cluster size. Then, the mean ReHo or FC strength in each significant cluster was extracted in each subject. A *post hoc t*-test was performed to find the detailed between-group ReHo or FC difference in each cluster employing the SPSS 19.0 software. Multiple comparison correction, i.e., the Bonferroni correction principle, was performed for *post hoc* comparisons. Finally, Pearson correlation analyses were performed between the mean ReHo or FC strength in each cluster and the cognitive test scores in the WMH with CI group using the SPSS 19.0 software with significance at *P* < 0.05.

## Results

### Demographic and Neuropsychological Data

As shown in Table 1, no significant differences in age, education and gender were found among the control group, the WMH without CI group and the WMH with CI group. The WMH with CI group performed significantly worse in MMSE, MoCA, TMT-B, Stroop-B, and Stroop-C tests than both the control group and the WMH without CI group (all *P* < 0.05). No significant differences in cognitive test scores were shown between the WMH without CI group and the control group.

**TABLE 1:** Demographic, neuropsychological and volume data.

**Items**	**Control (*n* = 33)**	**WMH without CI (*n* = 14)**	**WMH with CI (*n* = 14)**	**F or χ^2^**	***P*-value**
Age (years)	62.03±7.53	63.75±8.29	66.00±5.13	1.85	0.190
Education (years)	10.88±3.49	10.31±3.89	9.93±2.89	0.76	0.518
Gender (male: female)	16:17	8:6	7:7	0.30	0.861
MMSE	28.47±1.49	28.37±1.32	$26.86\pm 2.66{}^{a,b}$	–	**0.034**
MoCA	26.41±2.30	25.48±2.38	$20.43\pm 2.71{}^{a,b}$	–	**<0.001**
TMT-A	49.52±15.87	50.81±22.99	66.69±32.71	–	0.092
TMT-B	82.26±28.86	110.85±64.01	$155.90\pm 81.78{}^{a,b}$	–	**0.001**
Stroop-A	17.83±6.11	17.19±5.17	22.27±6.56	–	0.079
Stroop-B	19.97±7.12	20.75±6.79	$33.55\pm 19.00{}^{a,b}$	–	**0.015**
Stroop-C	29.96±7.89	30.73±11.92	$52.27\pm 36.68{}^{a,b}$	–	**0.007**
Total WMH volume (ml)	1.28±1.12	$6.06\pm 2.27{}^{a}$	$6.60\pm 4.25{}^{a}$	37.85	**<0.001**
Number of WMH lesions	8.81±3.53	$16.17\pm 6.02{}^{a}$	$15.43\pm 4.29{}^{a}$	20.12	**<0.001**
Whole brain volume (ml)	1239.62±185.18	1230.48±169.59	1246.71±168.05	0.12	0.89
Gray matter volume (ml)	483.74±75.80	483.63±72.96	497.57±62.44	0.21	0.721
White matter volume (ml)	432.87±78.33	424.62±69.04	440.21±63.28	0.29	0.788
Left hippocampal volume (ml)	2.77±0.39	2.53±0.38	2.58±0.28	1.42	0.283
Right hippocampal volume (ml)	2.71±0.55	2.52±0.48	2.68±0.35	0.92	0.408
Mean FD (mm)	0.17±0.05	0.15±0.04	0.18±0.07	1.00	0.376

*Values are presented as mean ± stand deviation (SD). One-way ANOVA was applied in the analyses of age, education and volume data. χ^2^ test was applied in the analysis of gender. The Kruskal–Wallis test was applied in the analyses of neuropsychological data. *P*-value < 0.05 appears in bold. ^*a*^*P* < 0.05, differs from the control group; ^*b*^*P* < 0.05, differs from the WMH without CI group. CI, cognitive impairment; FD, framewise displacement; MMSE, Mini Mental State Examination; MoCA, Montreal Cognitive Assessment; TMT-A and TMT-B, Trail Making Tests-A and B; WMH, white matter hyperintensities.*

### Volume Data

As shown in Table 1, both the WMH with CI group and the WMH without CI group displayed significantly larger total WMH volume and more number of WMH lesions than the control group (all *P* < 0.05). Notably, no significant differences in total WMH volume and the number of WMH lesions were found between the WMH with CI group and the WMH without CI group. Furthermore, no significant differences in whole brain volume, gray matter volume, white matter volume, bilateral hippocampal volume and mean FD were shown among the three groups.

### ReHo Data

**FIGURE 2:**
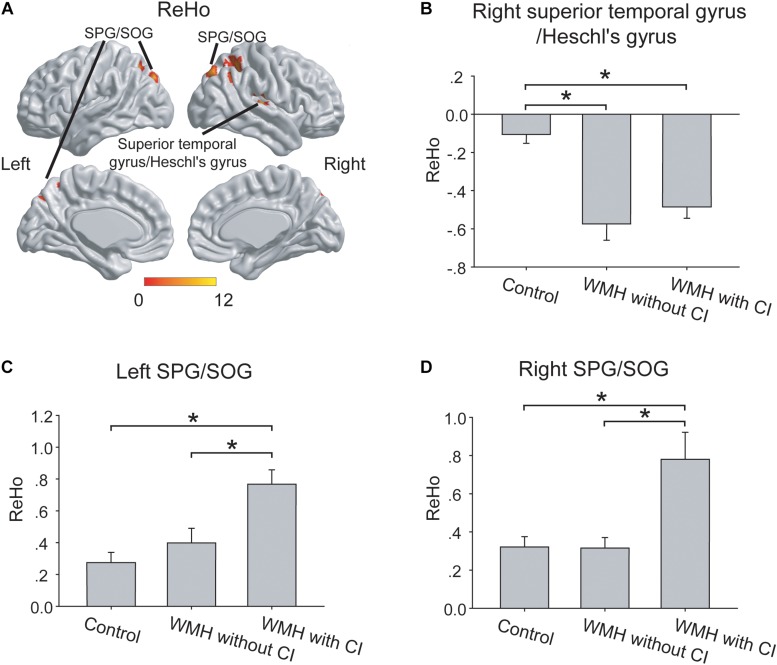
The group differences of ReHo. **(A)** The group differences of ReHo were shown in the right superior temporal gyrus/Heschl’s gyrus, left SPG/SOG and right SPG/SOG. **(B)** In the right superior temporal gyrus/Heschl’s gyrus, both the WMH with CI group and the WMH without CI group displayed lower ReHo than the control group. **(C,D)** In the left SPG/SOG and right SPG/SOG, the WMH with CI group showed higher ReHo than both the WMH without CI group and the control group. ReHo values have been normalized by subtracting the mean voxel-wise ReHo obtained for the entire brain, and then dividing the resultant value by the standard deviation. The thresholds were set at a corrected *P* < 0.01, determined by Monte Carlo simulation for multiple comparisons (voxel-wise *P* < 0.01, FWHM = 6.9 mm, cluster size > 1782 mm^3^). The color bars are presented with *F*-values. ^*^P < 0.05, CI, cognitive impairment; ReHo, Regional Homogeneity; SPG, superior parietal gyrus; SOG, superior occipital gyrus; WMH, white matter hyperintensities.

As shown in Figure 2A, the three groups displayed significant differences of ReHo in right superior temporal gyrus/Heschl’s gyrus, left superior parietal gyrus (SPG)/superior occipital gyrus (SOG) and right SPG/SOG.

#### *Post hoc* Analysis

In the right superior temporal gyrus/Heschl’s gyrus, both the WMH with CI group and the WMH without CI group displayed lower ReHo than the control group (both *P* < 0.001), and no significant difference of ReHo was shown between the 2 WMH groups (*P* = 0.27) (Figure 2B). Interestingly, in the left SPG/SOG and right SPG/SOG, the WMH with CI group showed higher ReHo than both the WMH without CI group (*P* = 0.002 and *P* < 0.001, respectively) and the control group (both *P* < 0.001) (Figures 2C,D).

### FC Data

Since the 2 WMH groups displayed significant differences of ReHo in the left SPG/SOG and right SPG/SOG, we further investigated the differences of FC pattern of the two regions among the three groups.

**FIGURE 3:**
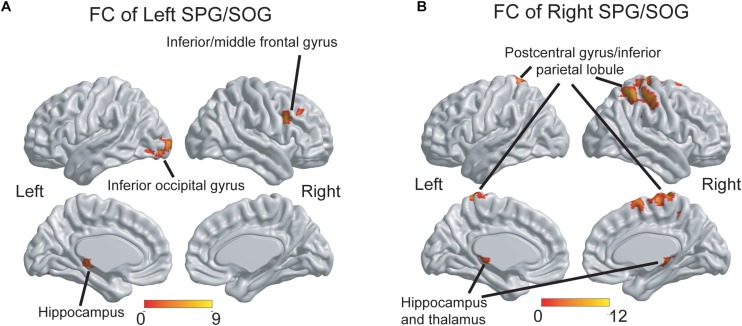
The group differences of FC patterns of the bilateral SPG/SOG. **(A)** Significant group differences of FC of the left SPG/SOG were shown in frontal, temporal and occipital regions. **(B)** Significant group differences of FC of the right SPG/SOG were shown in parietal, temporal, and thalamus regions. The thresholds were set at a corrected *P* < 0.01, determined by Monte Carlo simulation for multiple comparisons (voxel-wise *P* < 0.01, FWHM = 7.4 mm, cluster size > 2160 mm^3^). The color bars are presented with *F*-values. FC, functional connectivity; SPG, superior parietal gyrus; SOG, superior occipital gyrus.

As shown in Figure 3A, the three groups displayed significant differences of FC of the left SPG/SOG in the right inferior/middle frontal gyrus, left inferior occipital gyrus and left hippocampus. As shown in Figure 3B, the three groups displayed significant differences of FC of the right SPG/SOG in the bilateral postcentral gyrus/inferior parietal lobule and bilateral hippocampus and thalamus. The detailed coordinate information of above regions was shown in Table 2.

**TABLE 2:** Brain regions with group differences of ReHo or FC.

**Items**	**Brain regions with group differences**	**BA**	**Peak MNI coordinates x, y, z (mm)**	**Peak *F*-value**	**Cluster size (mm^3^)**
**ReHo**					
	Right superior temporal gyrus/Heschl’s gyrus	48	42, -51, 39	10.98	2079
	Left SPG/SOG	5, 7	−18, −60, 51	9.52	2268
	Right SPG/SOG	7, 19	24, −68, 36	11.15	1944
**FC of Left SPG/SOG**					
	Right inferior/middle frontal gyrus	44, 48	43, 14, 30	8.59	2430
	Left inferior occipital gyrus	19	−38, −79, −14	7.95	2862
	Left hippocampus	37	−24, −27, −6	8.10	2241
**FC of Right SPG/SOG**					
	Bilateral postcentral gyrus/inferior parietal lobule	1–7, 40	9, −33, 75	11.67	14634
	Bilateral hippocampus and thalamus	27	−14, −35, 9	8.23	2295

*The thresholds were set at a corrected *P* < 0.01, determined by Monte Carlo simulation for multiple comparisons (voxel-wise *P* < 0.01, for ReHo analysis, FWHM = 6.9 mm, cluster size > 1782 mm^3^, and for FC analysis, FWHM = 7.4 mm, cluster size > 2160 mm^3^). BA, Brodmann’s area; MNI, Montreal Neurological Institute; FC, functional connectivity; SPG, superior parietal gyrus; SOG, superior occipital gyrus.*

*Post hoc* analysis: As shown in Table 3, first, for the FC of the left SPG/SOG, the WMH with CI group displayed higher FC than the control group in all regions with significances (all *P* < 0.001). In contrast, the WMH without CI group displayed higher FC than the control group only in the left hippocampus (*P* = 0.004). Notably, the WMH with CI group showed higher FC than the WMH without CI group in the right inferior/middle frontal gyrus (*P* < 0.001). Second, for the FC of the right SPG/SOG, the WMH with CI group displayed higher FC than the control group in all regions with significances (*P* < 0.001 for the bilateral postcentral gyrus/inferior parietal lobule, and *P* = 0.002 for the bilateral hippocampus and thalamus). Compared with the control group, the WMH without CI group displayed higher FC in the bilateral hippocampus and thalamus (*P* = 0.003). Notably, the WMH with CI group displayed higher FC than the WMH without CI group in the bilateral postcentral gyrus/inferior parietal lobule (*P* < 0.001).

**TABLE 3:** FC data.

**Seed**	**Brain regions**	**FC strength**			***F***	***P*-value**
		**Control**	**WMH without CI**	**WMH with CI**		
**Left SPG/SOG**						
	Right inferior/middle frontal gyrus	0.21±0.16	0.15±0.15	$0.46\pm 0.11{}^{a,b}$	12.07	<0.001
	Left inferior occipital gyrus	0.31±0.24	0.39±0.19	$0.52\pm 0.25{}^{a}$	6.74	0.005
	Left hippocampus	0.12±0.13	$0.27\pm 0.15{}^{a}$	$0.28\pm 0.19{}^{a}$	7.05	0.002
**Right SPG/SOG**						
	Bilateral postcentral gyrus/inferior parietal lobule	0.41±0.11	0.40±0.14	$0.69\pm 0.18{}^{a,b}$	12.31	<0.001
	Bilateral hippocampus and thalamus	0.06±0.15	$0.21\pm 0.15{}^{a}$	$0.21\pm 0.18{}^{a}$	7.18	<0.001

*The thresholds were set at a corrected *P* < 0.01, determined by Monte Carlo simulation for multiple comparisons (voxel-wise *P* < 0.01, for ReHo analysis, FWHM = 6.9 mm, cluster size > 1782 mm^3^, and for FC analysis, FWHM = 7.4 mm, cluster size > 2160 mm^3^). ^*a*^*P* < 0.05, differs from the control group; ^*b*^*P* < 0.05, differs from the WMH without CI group. BA, Brodmann’s area; CI, cognitive impairment; MNI, Montreal Neurological Institute; FC, functional connectivity; SPG, superior parietal gyrus; SOG, superior occipital gyrus.*

### Behavioral Significance of ReHo and FC Alterations

**FIGURE 4:**
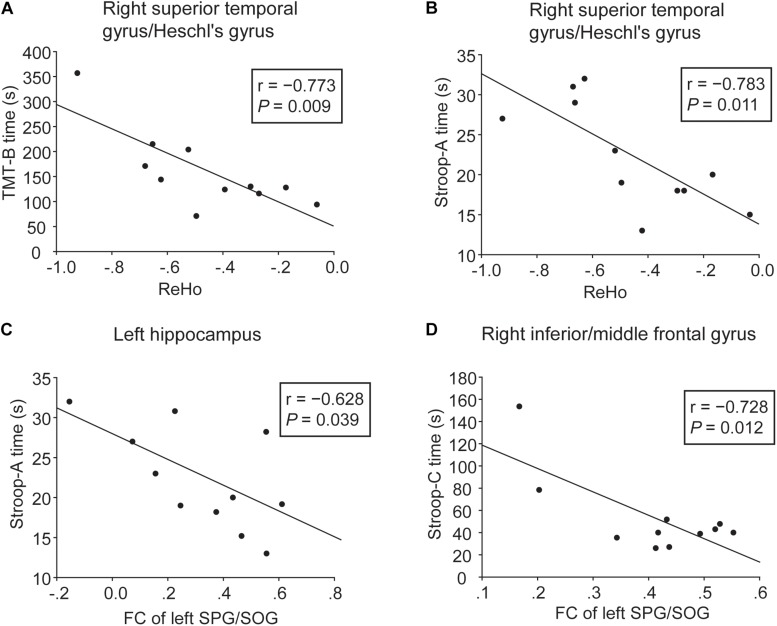
Correlation analyses between cognitive function and ReHo or FC in the WMH with CI group. **(A,B)** Significant negative correlation was shown between cognitive performances (TMT-B and Stroop-A) and ReHo in the right superior temporal gyrus/Heschl’s gyrus. **(C,D)** Significant negative correlation was shown between cognitive performances (Stroop-A and Stroop-C) and FC of the left SPG/SOG with the left hippocampus and the right inferior/middle frontal gyrus. Three WMH with CI subjects failed to perform these tests due to subjective unwillingness or hypopsia.

Correlative analyses between functional brain alterations and cognition were performed in the WMH with CI group. As shown in Figures 4A,B, lower ReHo in the right superior temporal gyrus/Heschl’s gyrus was significantly associated with longer TMT-B and Stroop-A time (*r* = −0.773, *P* = 0.009 and *r* = −0.783, *P* = 0.011, respectively), i.e., worse executive function and processing speed. As shown in Figures 4C,D, higher FC of the left SPG/SOG with the left hippocampus and the right inferior/middle frontal gyrus was significantly associated with shorter Stroop-A and Stroop-C time, respectively (*r* = −0.628, *P* = 0.039 and *r* = −0.728, *P* = 0.012, respectively), suggesting a compensatory effect.

## Discussion

The present study was the first to show the differences in ReHo and FC patterns between WMH with and without CI subjects who had comparable WMH burden. Worse global function, executive function and processing speed were shown in the WMH with CI group. The 2 WMH groups showed no significant differences in brain volume data. However, the WMH with CI group displayed higher ReHo in the bilateral SPG/SOG than the WMH without CI group. The WMH with CI group also displayed higher FC of the SPG/SOG with parietal and frontal regions. Furthermore, the ReHo and FC alterations were correlated with cognitive function in WMH with CI subjects.

A previous resting-state fMRI study investigated FC patterns in both WMH subjects with normal cognition and WMH subjects with CI, and found altered FC of posterior cingulate cortex with extensive regions in WMH subjects with CI. However, the WMH burden was not evaluated in the two groups (Sun et al., 2011). WMH is thought to disrupt white matter tracts and result in reorganization of functional brain patterns (Reijmer et al., 2015; De Marco et al., 2017). In the present study, since the 2 WMH groups had comparable WMH burden, the differences of functional patterns (i.e., ReHo and FC patterns) between the two groups could not be due to the difference of WMH burden, but could be related to the cognitive differences between the two groups. In the present study, higher ReHo in the SPG/SOG and higher FC of the SPG/SOG with parietal and frontal regions happened in the WMH with CI group, suggesting that the CI could be related to higher local coherence of activities in the SPG/SOG and higher connectivity between the SPG/SOG and parietal and frontal regions. Furthermore, the correlative analyses confirmed the results above.

In the present study, the WMH with CI group displayed poor performances in TMT-B, Stroop-B and Stroop-C tests, and increased ReHo in the SPG/SOG, suggesting that regional activities in parietal and occipital cortex were related to executive function in WMH with CI subjects. Parietal and occipital cortex plays a major role in the maintenance of normal cognition, including decision making, working memory, spatial updating and sensory attention (Medendorp et al., 2007; Tuladhar et al., 2007; Sulpizio et al., 2016), most of which are related to executive function. Furthermore, the maintenance of brain function relies on multiple brain areas that connect and interact with each other to serve different functions (Power et al., 2011). A balance between regional specialization and global integration is of vital importance for brain function (Tononi et al., 1998). A recent study found decreased FC of the default mode network and central executive network in subjects with both WHM and dementia (Kim et al., 2016). Another study investigated posterior cingulate cortex connectivity in WMH subjects with vascular cognitive impairment, no dementia, and showed both decreased FC with extensive regions and increased FC with parietal and temporal regions (Sun et al., 2011). In the present study, higher FC between the SPG/SOG and parietal and frontal regions were shown in the WMH with CI group. Higher FC of the left SPG/SOG with frontal regions were significantly associated with less worse executive dysfunction in WMH with CI subjects, suggesting a compensatory effect.

The mechanisms underlying the link between the functional alterations and CI in WMH subjects could be explained with a prominent cognitive model named “the scaffolding theory of aging and cognition (STAC)” (Reuter-Lorenz and Park, 2014). According to the STAC, cognitive decline is a consequence of “neural degradation,” “compensatory scaffolding,” and life-course factors. The neural degradation is thought to cause cognitive decline and is categorized as “neural challenges” and “functional deterioration.” The former refers to structural changes in the brain, including white matter damages (Reuter-Lorenz and Park, 2014), and the latter is indicators of maladaptive brain activity such as dedifferentiation of activities in visual areas (Park et al., 2004; Voss et al., 2008). In the present study, although the WMH with CI group and the WMH without CI group had comparable WMH burden, only the WMH with CI group displayed higher ReHo, i.e., higher local coherence of activities, in the SPG/SOG. The higher local coherence of activities could be one of indicators of maladaptive brain activity, and suggest functional deterioration in the WMH with CI group. This could be one of inducing factors for CI in WMH subjects. On the other hand, these negative indices might induce the onset of compensatory scaffolding that refers to compensatory reallocation or recruitment of cognitive resources or supplementary neural circuitry (Greenwood, 2007; Cramer et al., 2011; Reuter-Lorenz and Park, 2014). Compensatory scaffolding counteracts or ameliorates the damage effect of neural degradation. The higher FC of the SPG/SOG with parietal and frontal regions in the WMH with CI group indicated enhanced functional communications between the SPG/SOG and other regions. The results of correlative analyses showed that the enhanced communications partly compensated for executive dysfunction in WMH with CI subjects. Thus, the higher ReHo in the SPG/SOG and the higher FC of the SPG/SOG with parietal and frontal regions could represent functional deterioration and compensatory scaffolding, respectively, during the development of CI in WMH subjects.

Most of previous fMRI studies on WMH subjects investigated the effects of WMH burden on brain activities or connectivity. A recent study found that WMH burden modulated brain connectivity in healthy subjects, i.e., high WMH burden was associated with increased FC of default mode network and salience network with temporal cortex and parietal cortex, respectively (De Marco et al., 2017). A study investigated the spatial associations of intrinsic connectivity contrast with WMH volume in elderly subjects, and found that significant associations were detected between intrinsic connectivity contrast of SOG and WMH volume in subcortical white matter (Shi et al., 2017). A task fMRI study on healthy aging showed that greater WMH volume was associated with increased frontal activation and decreased frontal FC during performing a spatial search task (Lockhart et al., 2015). All these findings supported altered functional brain patterns across frontal, parietal, temporal and occipital regions in WMH subjects and also suggested a compensatory functional enhancement underlying the maintenance of normal cognition in WMH subjects. Consistent with these findings, the present study confirmed altered regional activities in parietal, occipital and temporal regions and increased FC with frontal, parietal and temporal regions in WMH subjects. Notably, the present study was performed on both WMH with and without CI subjects. The WMH without CI group displayed decreased ReHo in temporal regions and increased FC of the SPG/SOG with hippocampus. The increased FC with hippocampus might reflect a compensatory functional enhancement. In addition, compensatory functional enhancements were also shown in WMH with CI subjects. Thus, the present findings extended the compensatory functional enhancements on WMH subjects with mild CI.

Some limitations should be addressed. First, the sample size in the present study is small, especially for WMH subjects, and 3 WMH with CI subjects even failed to perform some of neuropsychological tests. The findings should be validated in a larger sample. We are continuing to recruit new participants with WMH to validate our findings. Second, due to the small sample size and a large number of correlation analyses between functional brain alterations and cognition, the results of correlative analyses would lose significance after correcting for the Bonferroni correction principle. Thus, the present findings should be treated with caution.

## Conclusion

In conclusion, with comparable WMH burden, WMH subjects with CI have higher local coherence of activities in the SPG/SOG and higher connectivity of the SPG/SOG with parietal and frontal regions than WMH subjects without CI. The findings provide novel insights into the functional alterations underlying the cognitive variety in WMH subjects and shed light on the investigation of surrogate markers for CI in WMH subjects.

## Ethics Statement

This study was carried out in accordance with the recommendations of the Drum Tower Hospital Research Ethics Committee. All subjects gave written informed consent in accordance with the Declaration of Helsinki. The protocol was approved by the Drum Tower Hospital Research Ethics Committee.

## Author Contributions

YX designed the study and revised the manuscript. QY and XC carried out the data collection and data analysis, and wrote the manuscript. RQ, LH, DY, and RL carried out the data collection. FB and BZ discussed the study. All authors approved the final version of the manuscript.

## Conflict of Interest Statement

The authors declare that the research was conducted in the absence of any commercial or financial relationships that could be construed as a potential conflict of interest.

## Footnotes

1 www.statistical-modeling.de/lst.html

2 http://www.fil.ion.ucl.ac.uk/spm

3 http://restfmri.net/forum/index.php

4 http://rfmri.org/DPABI
